# Distinguishing potential bacteria-tumor associations from contamination in a secondary data analysis of public cancer genome sequence data

**DOI:** 10.1186/s40168-016-0224-8

**Published:** 2017-01-25

**Authors:** Kelly M. Robinson, Jonathan Crabtree, John S. A. Mattick, Kathleen E. Anderson, Julie C. Dunning Hotopp

**Affiliations:** 1 Institute for Genome Sciences, University of Maryland School of Medicine, Baltimore, MD USA; 2 Department of Microbiology and Immunology, University of Maryland School of Medicine, Baltimore, MD USA; 3 Greenebaum Cancer Center, University of Maryland School of Medicine, Baltimore, MD USA

**Keywords:** Microbiome, Cancer, Batch effects, Genome sequencing, Cancer-associated bacteria, *Acinetobacter*, *Pseudomonas*, Stomach adenocarcinoma, Acute myeloid leukemia

## Abstract

**Background:**

A variety of bacteria are known to influence carcinogenesis. Therefore, we sought to investigate if publicly available whole genome and whole transcriptome sequencing data generated by large public cancer genome efforts, like The Cancer Genome Atlas (TCGA), could be used to identify bacteria associated with cancer. The Burrows-Wheeler aligner (BWA) was used to align a subset of Illumina paired-end sequencing data from TCGA to the human reference genome and all complete bacterial genomes in the RefSeq database in an effort to identify bacterial read pairs from the microbiome.

**Results:**

Through careful consideration of all of the bacterial taxa present in the cancer types investigated, their relative abundance, and batch effects, we were able to identify some read pairs from certain taxa as likely resulting from contamination. In particular, the presence of *Mycobacterium tuberculosis* complex in the ovarian serous cystadenocarcinoma (OV) and glioblastoma multiforme (GBM) samples was correlated with the sequencing center of the samples. Additionally, there was a correlation between the presence of *Ralstonia* spp. and two specific plates of acute myeloid leukemia (AML) samples. At the end, associations remained between *Pseudomonas*-like and *Acinetobacter*-like read pairs in AML, and *Pseudomonas*-like read pairs in stomach adenocarcinoma (STAD) that could not be explained through batch effects or systematic contamination as seen in other samples.

**Conclusions:**

This approach suggests that it is possible to identify bacteria that may be present in human tumor samples from public genome sequencing data that can be examined further experimentally. More weight should be given to this approach in the future when bacterial associations with diseases are suspected.

**Electronic supplementary material:**

The online version of this article (doi:10.1186/s40168-016-0224-8) contains supplementary material, which is available to authorized users.

## Background

Given that bacteria live in and on our bodies, they have a large potential to affect human health. An estimated 15–20% of cancers worldwide are linked to viral, parasitic, or bacterial infections that were responsible for 1.5 million cancer deaths in 2008 [1]. The best-studied examples of cancer-associated infectious agents are hepatitis B virus (HBV), human papillomavirus (HPV), Epstein-Barr virus (EBV), human immunodeficiency virus (HIV), *Schistosoma haematobium*, and *Helicobacter pylori*. A subset of these viruses are known to integrate into the human genome [2, 3], while viruses, parasites, and bacteria can all promote cancer through other mechanisms [4].

Of the bacteria known to be associated with carcinogenesis, the mechanisms linking *H. pylori* to gastric carcinoma and gastric mucosa-associated lymphoid tissue (MALT) lymphoma are best understood [5]. In addition to *H. pylori*, many other microbes have been associated with various carcinomas [6]. For example, *S. haematobium* with bladder carcinoma [7], *Salmonella typhi* with gallbladder cancer [8], *Chlamydia pneumoniae* with lung cancer [9], *Bacteroides fragilis* [10, 11] and *Streptococcus bovis* [12] with colon cancer, and *Escherichia coli* [13] and *Fusobacterium* spp. [14–18] with colorectal cancer. Frequently, bacteria are thought to contribute to carcinogenesis through increased inflammation, which promotes DNA damage [19]. While most cancer-related bacteria are the dominant member of the microbiome, it is possible that rare members could cause driver mutations and/or that dominant members might be more abundant in tumors due to a favorable tumor microenvironment.

*S. haematobium* is a parasitic flatworm classified as a definite carcinogen [7, 20] and was one of the first associations identified between an infectious agent and cancer formation [21]. The flatworm lays its eggs in the bladder mucosa causing constant irritation and inflammation [22] that is exacerbated when some eggs cannot be excreted through the urine and become trapped in the tissue [23]. The ability of *S. haematobium* to increase inflammation [22–24], decrease apoptosis [20, 23], and increase cell proliferation [20, 23] are the reasons for its classification as a definite carcinogen.

*H. pylori* also increases host inflammation and was the first bacterial species to be considered a carcinogen by the International Agency for Research on Cancer [25]. *H. pylori* is present in 90% of non-cardia gastric cancer cases and 86% of gastric MALT lymphoma cases [1]. *H. pylori* can alter host signaling pathways [25] and methylation of host genes [26]. Infections by *H. pylori* strains containing the cytotoxin-associated gene (*cag*) pathogenicity island cause upregulation of the mitogenic signaling pathway through activation of the *c-fos* and *c-jun* proto-oncogenes [25, 27], in addition to altering a number of other pathways.

The bioinformatics pipeline PathSeq [28] was used to identify an increase in *Fusobacterium* sequences in data from DNA [14] and rRNA-depleted RNA [15] from colorectal cancer samples relative to normal samples, as well as a decrease of *Bacteroidetes* and *Firmicutes* phyla in these colorectal cancer samples [14]. These findings were subsequently confirmed with 16S rRNA gene analysis and quantitative PCR on a larger group of samples, as well as using FISH to visualize the bacteria within the tumor cells [14]. While not definitively demonstrating that a *Fusobacterium* sp. can cause cancer, these results prompted more consideration for using sequencing data to identify candidate bacteria involved in carcinogenesis, without relying on culture-based techniques.

As more genome sequencing data becomes available, secondary, retrospective studies can be carried out to test other hypotheses. However, such studies are not without biases since the analysis is conducted looking through a lens that can often be clouded with uncertainty related to sequencing type, unknown metadata factors, and lack of access to original samples. For example, bias can be introduced from the type of sequencing undertaken, which is not always clear in associated metadata or text on methods in publications. For example, human RNA sequencing (RNA-Seq) data is often from a library constructed from poly-A-selected RNA, which removes the RNA of many but not all bacteria. Therefore, it is impossible to determine which bacteria may be present in the sample, but missing from poly-A-selected data. For example, The Cancer Genome Atlas (TCGA) analysis of the poly-A-selected RNA-Seq stomach adenocarcinoma (STAD) data detected *H. pylori* only sporadically, which they attribute to either the decline of bacterial abundance upon progression from chronic gastritis to subsequent carcinoma or the technical loss of luminal bacteria during specimen processing [29].

Using a method analogous to PathSeq, we previously presented evidence supporting the presence of various microbes in tumor samples from TCGA. This study predominantly focused on identifying bacteria-human lateral gene transfer (LGT) events in a subset of TCGA data (Table 1). Putative bacteria-human LGT events were found in tumor suppressor and proto-oncogenes in stomach adenocarcinoma samples, as well as in the mitochondrial genome of acute myeloid leukemia samples (AML) [30]. In these cases, it was reported that the microbiome-associated bacteria of the samples with putative LGTs were highly reflective of the bacterial species from which the putative LGT originated [30]. Specifically, the STAD samples with putative *Pseudomonas*-like integrations had *Pseudomonas* spp. as the predominant member of their microbiome, while the STAD samples lacking LGT had a higher relative abundance of *Enterobacteriaceae* [30]. Also, the AML samples with putative *Acinetobacter*-like DNA integrations had an increased relative abundance of microbiome-associated *Acinetobacter* sequences [30].

**Table 1:** List of cancer types analyzed, their TCGA designated abbreviations, and the sequencing type analyzed

Cancer type	Abbreviation	Sequencing type^1^
Acute myeloid leukemia	AML	RNA-Seq (192)
Breast cancer	BRCA	RNA-Seq (451)
Glioblastoma multiforme	GBM	RNA-Seq (1), DNA (10), WGA (147)
Kidney clear cell carcinoma	KIRC	RNA-Seq (420)
Kidney papillary carcinoma	KIRP	RNA-Seq (15)
Lung adenocarcinoma	LUAD	RNA-Seq (76)
Lung squamous cell carcinoma	LUSC	RNA-Seq (174)
Ovarian serous cystadenocarcinoma	OV	WGA (310), DNA (13)
Stomach adenocarcinoma	STAD	RNA-Seq (71)

^1^ As denoted by the TCGA barcode, the sequencing types are RNA-Seq, DNA, or whole genome amplified (WGA). The number of samples analyzed for each sequencing type is denoted in the parentheses and is not the number of samples where bacterial read pairs were identified

We sought to more systematically examine the bacterial sequences detected across numerous sample types in TCGA data and to develop techniques to distinguish the presence of biologically/medically significant microbes from the presence of bacterial contamination, which can arise in numerous ways. For example, the presence of bacterial DNA in laboratory kits and reagents was found to impact the results of 16S rRNA gene and shotgun metagenomics methods, particularly samples with a low microbial biomass [31]. Microbial contamination has been identified in nucleic acid extraction spin columns, where column contamination during manufacturing was responsible for the discovery of a novel DNA virus [32]. Another example of microbial contamination in a human genomics project was the identification of HPV-18 in cervical cancer and nine other cancer types from TCGA [33]. This was determined to be contamination in the non-cervical cancer samples when it was realized that the HPV-18 reads had characteristics of the typical HPV-18 integration in HeLa cells and lacked reads aligning to the regions of the HPV-18 genome that are typically not integrated [33]. Therefore, the presence of HPV-18 in the non-cervical cancer TCGA data suggests that HeLa cells which contain this HPV-18 integration were actually the cause of the contamination [33]. Additionally, contamination from liver cancer samples was the source of HBV in one kidney cancer TCGA sample [34].

Here, we present the results of our analysis of an early release of TCGA data for evidence of bacteria. This examination reveals that some bacterial sequences can be attributed to systematic contamination of all samples or to batch effects likely associated with contamination, while other bacteria are associated with particular tumor types without an obvious source of contamination. Since we cannot completely rule out contamination as a source of these sequences, these bacteria-tumor associations should bear closer scrutiny in future studies aimed at examining the roles bacteria might play in oncogenesis.

## Results

### Bacterial presence in TCGA data

In order to determine the microbial component of various cancers, we analyzed the relative abundance (subsequently referred to as abundance) of bacteria-derived paired-end Illumina sequencing in TCGA data that was made available in the Sequence Read Archive (SRA), as previously described [30]. Briefly, reads were aligned to the human genome and RefSeq with Burrows-Wheeler aligner (BWA) ALN/SAMPE, a likely taxonomic assignment was made for each read pair (Additional file 1: Figure S1), and the results (Additional file 2) were loaded into a database for analysis and visualization with Krona [35] and MicroView, which can be found at http://microview.igs.umaryland.edu/tcga_v1/. MicroView is an interactive website and derivative of our LGTview [30], which enables the interrogation of metadata using a graphical interface connected to an underlying database of raw data for each bacterial read pair (Additional file 2). MicroView is referenced in the remainder of the manuscript to denote when data was mined from MicroView that could not be represented in an individual figure. An operational taxonomic unit (OTU) is assigned to each read and read pair that should be considered approximate, as the assignment is likely to be influenced by the genomes in RefSeq. Therefore, for example, *Pseudomonas* reads should be considered *Pseudomonas*-like reads.

**Fig. 1:**
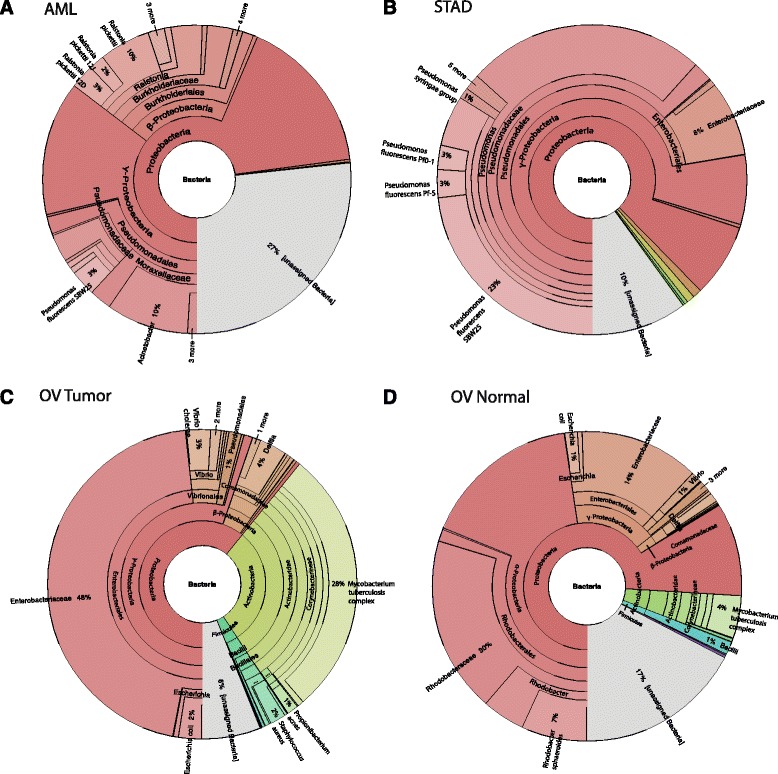
The proportion of read pairs with each bacterial operational taxonomic unit (OTU) is illustrated with Krona plots for AML samples (**a**), STAD samples (**b**), OV tumor samples (**c**), and OV normal samples (**d**). While the STAD samples seem to be almost entirely *Pseudomonas* spp., the AML samples show some *Pseudomonas* spp., *Acinetobacter* spp., and *Ralstonia* spp. The OV samples show evidence of *Mycobacterium* spp., as well as *Enterobacteriaceae*. There is an increased abundance of *Mycobacterium* spp. in the 154 OV tumor samples and *Rhodobacteraceae* spp. in the 156 OV normal control samples

Nine cancer types were examined (Table 1) and found to have differing abundances of putative bacterial read pairs ranging from 11% of patients with at least one sample containing bacterial read pairs in lung squamous cell carcinoma (LUSC) to 100% of patients having at least one sample with bacterial read pairs in STAD (Table 2). STAD, AML, and ovarian serous cystadenocarcinoma (OV) have large proportions of *Pseudomonas*, *Acinetobacter*, and *Mycobacterium* read pairs, respectively (Fig. 1), which were not frequently identified in other cancer types (MicroView). Despite different locations in the human body, read pairs from particular bacterial taxa were repeatedly present in most cancer types (e.g., *Enterobacteriaceae*, *Propionibacterium*, *Ralstonia*, and *Staphylococcus* taxa) (MicroView).

**Table 2:** Summary of cancer types and bacterial presence

Cancer type	Total no. of seq. runs	Seq. runs with any bacterial read pair	Total no. of patients	Patients with any bacterial read pair	Samples^1^			Samples with any bacterial read pair		
					Tumor	Normal		Tumor	Normal	
						Solid tissue	Blood		Solid tissue	Blood
AML	553	197 (36%)	187	117 (63%)	192	0	0	117 (61%)	NA^2^	NA
BRCA	754	90 (12%)	430	90 (21%)	425	26	0	87 (20%)	3 (12%)	NA
GBM	1476	600 (41%)	83	72 (87%)	78	0	78	68 (87%)	NA	59 (76%)
KIRC	647	72 (11%)	401	72 (18%)	398	22	0	72 (18%)	0 (0%)	NA
KIRP	15	9 (60%)	15	9 (60%)	15	0	0	9 (60%)	NA	NA
LUAD	76	10 (13%)	76	10 (13%)	76	0	0	10 (13%)	NA	NA
LUSC	306	20 (7%)	174	20 (11%)	172	2	0	20 (12%)	0 (0%)	NA
OV	1991	561 (28%)	160	127 (79%)	154	20	136	105 (68%)	15 (75%)	93 (68%)
STAD	143	142 (99%)	71	71 (100%)	71	0	0	71 (100%)	NA	NA

^1^ There can be multiple tumor and normal samples for each patient. A sample is defined as each tumor or normal sample. It should be noted that a tumor sample may have DNA and RNA extracted for sequencing and as such may result in more than one analysis per sample.

^2^NA = not applicable

### Dominant bacteria in TCGA sequencing projects

**Fig. 2:**
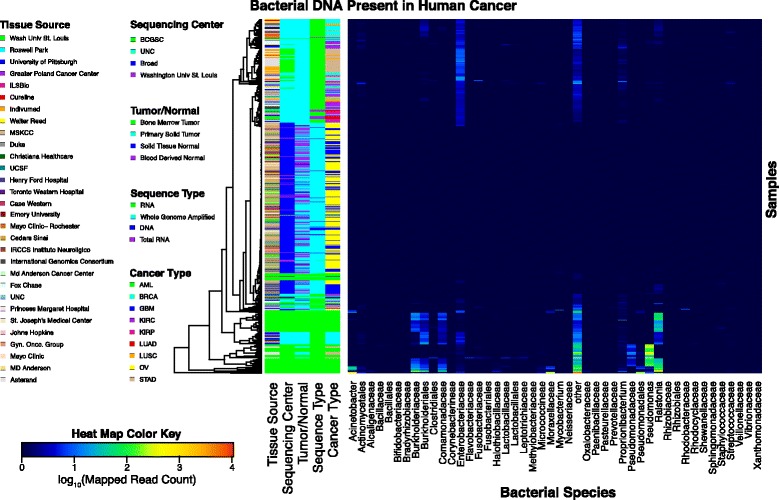
The normalized number of bacterial read pair counts for each sample is represented in this heat map for all bacterial taxa with >20 reads per one million total reads. The dendrogram represents the agglomerative hierarchical clustering of the samples based on their bacterial reads. Color-coded bars are present to represent the cancer type, tissue source site, sequencing type, sample type, and sequencing center. GBM and OV samples have the lowest abundance of bacterial taxa, while the only samples showing increased read counts of *Mycobacterium* spp. are OV samples. *Enterobacteriaceae* and “other” were the dominant taxa in most cancer types, except for *Ralstonia* spp. in GBM, *Ralstonia* spp. and “other” in AML, and *Enterobacteriaceae* and *Mycobacterium* spp. in OV

All of the cancer types investigated had one or more samples containing read pairs present that were assigned to bacterial taxa (Table 2). However, only STAD and kidney papillary carcinoma (KIRP) had more sequencing runs with bacterial read pairs than sequencing runs without any bacterial read pairs present (Table 2, Additional file 3: Figure S2). Some bacterial species were the dominant taxa of a specific cancer type, as was the case with *Pseudomonas* in STAD, *Acinetobacter* in AML, and *Mycobacterium tuberculosis* complex in OV tumor samples (Fig. 1). In the STAD samples, 62% of the bacterial read pairs were assigned as *Pseudomonas* (Fig. 1), although most of the read pairs are found in only a few samples (Fig. 2). In contrast, the AML samples were dominated by 7% *Pseudomonas*, 10% *Acinetobacter*, and 15% *Ralstonia* read pairs (Fig. 1). Although kidney clear cell carcinoma (KIRC) also had *Pseudomonas* read pairs present in the samples, it was responsible for 10% of the total bacterial read pairs (MicroView). The OV samples surprisingly revealed evidence of *Mycobacterium* read pairs, as well as *Enterobacteriaceae* read pairs (Fig. 1). The main difference in bacterial proportions when comparing the tumor and healthy tissue samples for OV is the increased abundance of *Mycobacterium* read pairs in the tumor samples, as well as the presence of *Rhodobacteraceae* read pairs in the normal samples (Fig. 1). A similar comparison for AML and STAD cannot be made, as no normal samples were available.

In general, samples of DNA and whole-genome amplified DNA were clustered together regardless of sequencing center or cancer type since samples were sequenced by both The Broad Institute of MIT and Harvard (Broad) and Washington University in St. Louis from OV and glioblastoma multiforme (GBM) (Fig. 2). DNA samples had a lower mapped read count relative to the RNA samples. However, while the DNA samples are in one distinct cluster, the RNA samples formed many distinct clusters that at times relate to cancer type (e.g., AML) (Fig. 2). However, AML samples had multiple distinct groups (Fig. 2).

### A comparison of aggregate bacteria found in tumor and normal samples

Only five sets of samples in the data set we used had tumor-normal pairs. Breast cancer (BRCA), OV, and GBM had both tumor and normal samples that contained bacterial sequences. In contrast, bacterial read pairs were only present in the tumor samples for KIRC and LUSC, although LUSC only had two normal samples sequenced in this release (Table 2). While the majority of the bacterial taxa in the BRCA tumor and non-paired normal samples were similar, there were some minor differences (MicroView). However, it is important to note that only 3 of 26 normal samples and 87 of 425 tumor samples contained bacterial read pairs at the time this data was downloaded and analyzed (Table 2). From the samples containing bacteria, the normal samples only had 4,526 bacterial read pairs compared to 983,611 bacterial read pairs from the tumor samples (Table 2). The GBM dataset had approximately the same number of bacterial read pairs per sample for both the tumor and paired normal samples (Table 2). Both the tumor and normal samples had about a third of the read pairs from *Ralstonia picketti* with low levels of read pairs from *Enterobacteriaceae*, *Pseudomonas aeruginosa*, and *M. tuberculosis* complex (Additional file 4: Figure S3). There was a shift in the proportion of *Propionibacterium acnes* read pairs with an ~10% increase in the aggregate for the normal GBM samples (Additional file 4: Figure S3). Additionally, there is a difference in the percentage of read pairs classified as “unassigned bacteria” between the GBM tumor and normal samples (Additional file 4: Figure S3). This may happen, in general, if the bacterial read pairs in one sample align to parts of conserved regions of bacterial genomes, thus making it more difficult to determine a specific taxon to assign to the read pair (Additional file 1: Figure S1). Overall, these tumor-normal pairs clustered together in the heat map, indicating their similarity (Fig. 2). Due to the similarity of the tumor and normal samples, in the remainder of this analysis, they will be grouped together by cancer type, except when explicitly noted.

### *Mycobacterium* spp. in samples

Upon evaluating the samples with read pairs that had assigned bacterial taxa, it was surprising that read pairs designated as *M. tuberculosis* complex could be found in both the tumor and paired-normal samples of OV and GBM (Fig. 1). In particular, bacteria are not expected in the GBM samples as this is a type of brain tumor and few bacteria can cross the blood-brain barrier. The taxonomic assignment of the *Mycobacterium* read pairs from the OV and GBM dataset was confirmed using a BLASTN search against NT, which is a more comprehensive database of reference sequences than what was used in the initial OTU assignment.

Five sequencing centers generated the data for all of the cancer types in our analysis. All of the sequencing centers with bacterial read pairs contained at least one sample with *Mycobacterium* spp. read pairs (MicroView). To compare results, we normalized by the total number of read pairs sequenced, using the log_10_-transformed ratio of *Mycobacterium* read pairs to total read pairs. A disproportionate number of OV samples sequenced at The Broad Institute had a log_10_ ratio of *Mycobacterium* read pairs to a total read pairs of ≥−7 (Table 3), meaning there was at least one *Mycobacterium* read pair for every ten million read pairs sequenced. The difference between samples sequenced at The Broad Institute and Washington University was considered significant by Fisher’s exact test (*p* value = 4.03 × 10^−7^). There was only one sample sequenced at Washington University in St. Louis that had such a large proportion of *Mycobacterium* read pairs, and none of the OV samples sequenced at Washington University in St. Louis contained these elevated levels of *Mycobacterium* spp. read pairs (Table 3).

**Table 3:** Relative *Mycobacterium* spp. read pairs and sequencing center for each sample

Cancer type	Sequencing centers																			
	Total samples^1^					Samples with bacteria					Samples with a log_10_ (*Myco*./total reads) ratio ≥ −7					Samples with a log_10_ (*Myco*./total reads) ratio ≥ −4				
	Baylor^2^	BCGSC	Broad	UNC	Wash.	Baylor	BCGSC	Broad	UNC	Wash.	Baylor	BCGSC	Broad	UNC	Wash.	Baylor	BCGSC	Broad	UNC	Wash.
AML	–^﻿3^﻿	192	–	–	–	–	117	–	–	–	–	33	–	–	–	–	0	–	–	–
BRCA	–	340	–	393	–	–	0	–	90	–	–	0	–	40	–	–	0	–	0	–
GBM	1	–	154	–	3	0	–	125	–	3	0	–	29	–	1	0	–	0	–	0
KIRC	–	397	–	248	–	–	0	–	72	–	–	0	–	26	–	–	0	–	0	–
KIRP	–	–	–	15	–	–	–	–	9	–	–	–	–	2	–	–	–	–	0	–
LUAD	–	58	–	18	–	–	0	–	10	–	–	0	–	3	–	–	0	–	0	–
LUSC	–	136	–	167	–	–	0	–	20	–	–	0	–	4	–	–	0	–	0	–
OV	3	–	262	–	97	0	–	197	–	20	0	–	106	–	0	0	–	1	–	0
STAD	–	71	–	–	–	–	71	–	–	–	–	22	–	–	–	–	0	–	–	–

^1^ Total samples and samples with bacteria are defined as an analyte from each sample, so if one tumor sample had DNA and RNA sequenced or had RNA sequenced at multiple centers, each analysis of each nucleic acid is counted individually. For example, while there were only 451 BRCA samples (Table 1), most samples were sequenced at two sequencing centers, resulting in a total of 733 entries in the “Total samples” column of this table.

^2^Baylor = Baylor College of Medicine Human Genome Sequencing Center; BCGSC = British Columbia Genome Sequencing Center; Broad = The Broad Institute; UNC = University of North Carolina, Chapel Hill; Wash. = Washington University in St. Louis.

^3^​Sequencing centers that did not sequence any samples of a specific cancer type are denoted by a ﻿"–"

The Broad Institute had a National Institute of Allergy and Infectious Diseases-funded white paper to sequence *Mycobacterium* spp. at approximately the same time that these TCGA samples would have been sequenced. Combined with the batch effects associated with the sequencing center, we conclude that the *Mycobacterium* reads most likely arose from contamination. This conclusion is similar to another contamination analysis of TCGA OV samples from The Broad Institute when dengue virus 2 was determined to be present in the sequence data due to cross-contamination from the sequencers [33]. A center that sequences many microbial genomes along with human genomes (like The Broad Institute) will have more contamination from those microbial genomes. A sequencing center that primarily sequences human samples would not have as much likelihood of experiencing these same microbial contamination issues but may have similar levels of contamination from other sources. However, overall, samples from The Broad Institute have the lowest levels of bacterial sequences when normalized for the amount of data sequenced (Fig. 2).

### Presence of *Enterobacteriaceae* in all cancer types

*Enterobacteriaceae* reads, which were present in most cancer types (Fig. 2), may be a result of *E. coli* DNA contamination in recombinant enzymes used in whole-genome amplification and library construction. Consistent with this, we previously identified junctions of *E. coli* and human DNA in the OV data examined here that we attribute to occurring during whole genome amplification of these samples [30]. Given these concerns and the presence of *E. coli* in all cancer types, we conclude that these sequences have a high chance of arising via contamination, and as such, we did not examine them further. However, it is important to note that *E. coli* has been associated with various forms of cancer [36], and we could be overlooking an important biological observation.

### Potential bacterial contaminants found in samples aggregated across cancer type

Read pairs from *Staphylococcus epidermidis*, *P. acnes*, and *Ralstonia* spp. were present collectively at low levels in all of the cancer types (Fig. 1, Additional file 4: Figure S3, Table 4, Additional file 2), suggesting they may be contaminants. *S. epidermidis* and *Propionibacterium* spp. are skin-associated bacteria and may contaminate reagents after being shed from the skin of a laboratory and manufacturing personnel. *Ralstonia* spp. is thought to be a common environmental contaminant in laboratories [31].

**Table 4:** Number of *Propionibacterium* spp., *Ralstonia* spp., and *Staphylococcus epidermidis* read pairs in all cancer types

Cancer type	Total read pairs	Total bacterial read pairs	*Propionibacterium* read pairs	*Ralstonia* read pairs	*Staphylococcus epidermis* read pairs
AML	7,955,502,437	29,458,420	46,372	4,502,338	9013
BRCA	6,093,925,360	988,137	14,526	78,895	1220
GBM	5,391,069,119	20,802	1976	8244	105
KIRC	5,070,366,679	356,674	31,553	5104	1428
KIRP	541,775,677	52,313	1394	669	128
LUAD	980,790,987	29,583	534	914	28
LUSC	1,166,304,426	107,515	12,403	5345	5714
OV	8,643,898,191	34,724	196	64	23
STAD	6,689,562,270	4,847,299	31,006	1983	1323

**Fig. 3:**
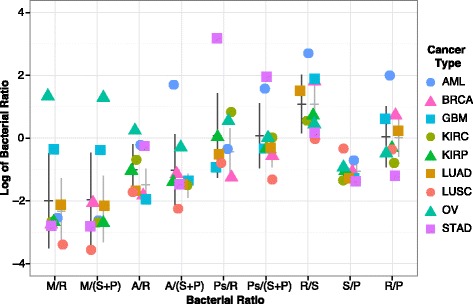
The log_10_-transformed ratios of bacterial read pairs are shown for all nine cancer types comparing counts of *Mycobacterium* (M), *Acinetobacter* (A), *Pseudomonas* (Ps), *Staphylococcus epidermidis* (S), *Ralstonia* (R), and *Propionibacterium* (P) read pairs. *Staphylococcus* and *Propionibacterium* were grouped together after an initial comparison found them to frequently have the same relative proportions. In addition, they are both commonly found on the human skin and may arrive in the samples through the same mechanism. The black horizontal lines represent the average of the log_10_-transformed ratios across all the cancer types. When points are clustered near the mean from all cancer types, it suggests a common source of the bacterial reads. Given the diverse cancer types and the numerous collection and sequencing centers, we interpret those contributions to be from a general source of contamination, whereas when a set of samples does not cluster with the others (e.g., OV and GBM in M/R), it suggests a more specific source of the bacterial sequences. The latter can be due either to contamination or a biological reason, which cannot be distinguished here. This was found for comparisons containing (a) *Mycobacterium* in OV and GBM, (b) *Pseudomonas* in STAD, and (c) *Acinetobacter*, *Ralstonia*, and *Pseudomonas* in AML. The standard deviations﻿ with all data, de﻿picted by the vertical black line﻿, of each of the bacterial comparisons are shown across all nine datasets. AML, OV, and STAD, which are cancer types that have at least one predominant bacterial species, not including *Enterobacteriaceae*, were excluded from a subsequent mean and standard deviation calculation, depicted by the gray horizontal and ﻿vertical lines, respectively﻿. Excluding AML, OV, and STAD decreases the standard deviation for the comparisons involving *Acinetobacter* spp. and *Pseudomonas* spp. This suggests that the presence of *Acinetobacter* spp. and *Pseudomonas* spp. may be attributed to similar levels of general contamination of all samples by a similar mechanism, except in AML, OV, and STAD

We sought to develop a metric to identify taxa associated with a common contaminant source. Using ratios, we compared the counts of bacterial taxa to that of commonly identified bacterial taxa in these data sets, including *Pseudomonas*, *Acinetobacter*, and *M. tuberculosis* complex (Fig. 3). Log ratios that cluster tightly near the mean, with a low standard deviation, are taken to represent taxa clustered from a common source of contamination across multiple tumor samples. For example, the log-ratio comparing *Staphylococcus* spp. and *Propionibacterium* spp. is consistently around one for every cancer type analyzed, which means there are ten times as many *Propionibacterium* reads as *Staphylococcus* reads. As *Staphylococcus* spp. and *Propionibacterium* spp. could both be present on the human skin, this poses one mechanism for co-contamination from these two bacteria. Outliers in this ratio-based analysis suggest a different phenomenon for the two samples, which might be an alternate form of contamination or might be biologically relevant. For example, and as discussed previously, *Mycobacterium* reads were over-represented when compared to *Staphylococcus* and *Propionibacterium* reads in OV, with moderate ratios in GBM (Fig. 3); this likely reflects *Mycobacterium* contamination at the sequence center level. Other outliers include *Acinetobacter* in AML, *Pseudomonas* in STAD, and *Ralstonia* in AML. While *Acinetobacter* reads are over-represented when compared to *Staphylococcus* reads and *Propionibacterium* reads, *Acinetobacter* reads do not appear to be over-represented compared to *Ralstonia* or *Pseudomonas* reads in AML (Fig. 3). This suggests that there may be a relationship between *Acinetobacter*, *Ralstonia*, and *Pseudomonas* taxa in AML.

When a relationship is expected between taxa, like *Staphylococcus* and *Propionibacterium*, a low standard deviation is observed, indicating that they may share a common mechanism of contamination. However, other sources of contamination can alter the standard deviation, obscuring this observation. To further examine the variation of these ratios across all nine cancer types to identify a possible common mechanism, the standard deviation was calculated for these ratios based on all nine cancers and compared to the standard deviation when outlier datasets were removed, namely AML, OV, and STAD. We initially identified these three cancer types as outliers in the ratio analysis, and more specifically, it was expected that the specific higher abundances of *Acinetobacter* reads, *Mycobacterium* reads, *Ralstonia* reads, and *Pseudomonas* reads caused higher standard deviations in those comparisons. The standard deviations do decrease upon the removal of AML, OV, and STAD (Fig. 3), and we interpret this to mean that low levels of *Acinetobacter* reads, *Ralstonia* reads, and *Pseudomonas* reads have a common source of contamination across all samples, while high levels may arise due to an alternate explanation, which could be an alternate source of contamination. Therefore, we sought to identify further sources of contamination for *Pseudomonas, Ralstonia,* and *Acinetobacter* reads.

### Examination of potential bacterial contaminants found in individual samples

**Fig. 4:**
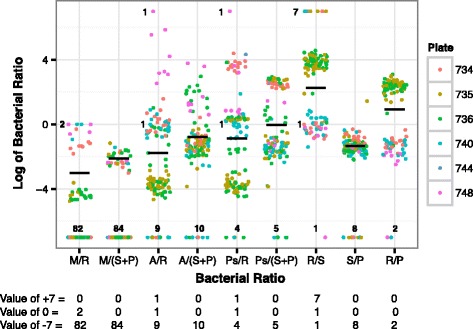
The log_10_-transformed ratios of bacterial read pairs from a specific taxon to the total number of bacterial read pairs from that patient were calculated for all AML samples as described in Fig. 3. Ratios where the numerator was 0 were assigned an arbitrary value of −7, ratios where both the numerator and denominator were 0 were assigned an arbitrary value of 0, and comparisons where the denominator was 0 were assigned an arbitrary value of 7. Counts for the number of patients where one of these arbitrary values were assigned are shown below the x-axis. When >5 points are at a given value, some data points may overlap each other making it impossible for all data points to be seen. The samples were all collected and sequenced at the same locations, and the data are color-coded by plate. The samples on plate 735 and 736 have a higher abundance of *Ralstonia* spp., suggesting that these samples may have been contaminated and this may be responsible for the bimodal distribution of samples in those comparisons. As was seen with the plot of all the cancer types, there is very little variation for the comparison of *Staphylococcus* spp. to *Propionibacterium* spp

In AML, as with all the cancer types, there is very little variation for the comparison of *Staphylococcus* reads to *Propionibacterium* reads (Fig. 4), which is expected for co-contaminants. In contrast, ratios including *Ralstonia* reads or *Pseudomonas* reads have a bi-modal distribution. Samples on plates 735 and 736 have a higher abundance of *Ralstonia* spp. (Fig. 4), suggesting that these plates may have been contaminated. Upon further examination of *Ralstonia* reads in other cancer types, there may also be a batch effect by plate in BRCA. For example, plates A056 and A084 appear distinct from plates A00Z, A034, A109, A10J, A115, and A12D (Additional file 5: Figure S4). Despite the relationship of *Ralstonia*, *Acinetobacter*, and *Pseudomonas* in the AML samples described above, there is no correlation between plate and either *Acinetobacter* or *Pseudomonas*. Therefore, we conclude that *Ralstonia* read pairs arose from a contamination that occurred at the plate level in AML while *Acinetobacter* and *Pseudomonas* read pairs result from either a separate contamination that could not be identified or from a biologically relevant source, like the microbiome. The initial correlation may have resulted from using a single average on the aggregate study containing such broad bi-modal distributions of individual samples.

**Fig. 5:**
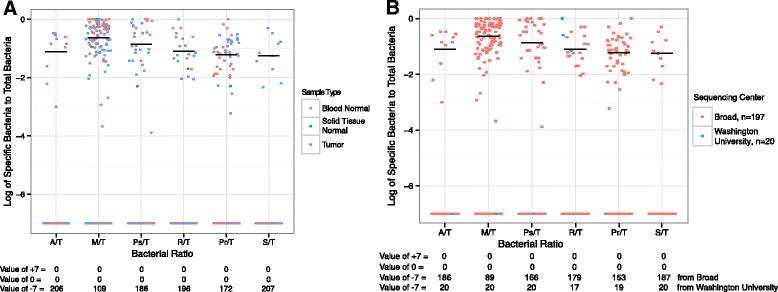
The log_10_-transformed ratios of bacterial read pairs from a specific taxon to the total number of bacterial read pairs from that patient were calculated for all ovarian cancer patients. These comparisons include counts of *Mycobacterium* (M), *Acinetobacter* (A), *Pseudomonas* (Ps), *Staphylococcus epidermidis* (S), *Ralstonia* (R), and *Propionibacterium* (P) read pairs and the total (T) number of bacterial read pairs from that patient. For example, A/T is the log_10_-transformed ratio of the number of *Acinetobacter*-like read pairs divided by the number of total bacteria-like read pairs. The black horizontal line represents the average of the log_10_-transformed ratios, after excluding those samples lacking data. The patients are color-coded by sample type (**a**) and by sequencing center (**b**). The number of samples from each sequencing center is denoted (**b**). Ratios where the numerator was 0 were assigned an arbitrary value of −7, ratios where both the numerator and denominator were 0 were assigned an arbitrary value of 0, and comparisons where the denominator was 0 were assigned an arbitrary value of 7. Counts for the number of patients where one of these arbitrary values were assigned are shown below the x-axis and are broken down by sequencing center when the sequencing center denotes the color (**b**). When >5 points are at a given value, some data points may overlap each other making it impossible for all data points to be seen

In the OV data set containing the *Mycobacterium* read pairs described above, we did not find any such associations with available metadata when examining samples individually (Fig. 5). Color-coding by patient or sample type revealed no obvious trend (Fig. 5a), while color-coding by sequencing center reveals that *Mycobacterium* reads are associated predominantly with just one sequencing center (Fig. 5b).

**Fig. 6:**
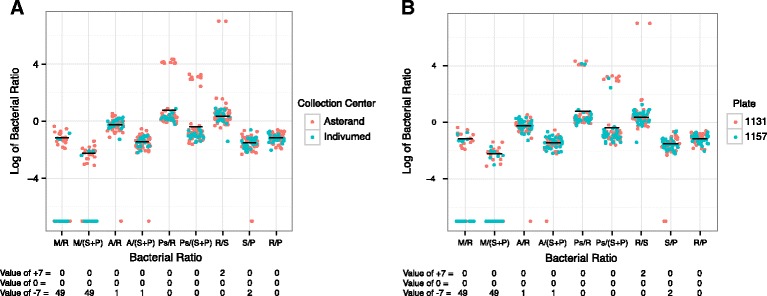
The log_10_-transformed ratios of bacterial counts by patient were calculated as described in Fig. 3 for stomach adenocarcinoma samples with the various panels color-coded by collection center (**a**) and plate (**b**). For each bacterial comparison, there is a mostly unimodal distribution indicating that there is little variation across samples. The ratios with *Pseudomonas* spp. have a few outliers from the Asterand collection center, but otherwise, no correlation between these factors and the relative abundance of the specific bacteria can be identified

In STAD, there appears to be no difference in bacterial abundances or ratios correlated with sequencing center, collection center, or plate when samples are examined individually (Fig. 6). The samples have a unimodal distribution for all of the comparisons, except for the comparisons with samples containing *Pseudomonas* reads where a small subset of samples collected from Asterand have an increased abundance of *Pseudomonas* read pairs, therefore separating them from the other samples. A more precise attribute to distinguish these samples from the others could not be determined. This indicates that there is little variation within the single sequencing center, collection center, and plate from which these samples were collected. As the plot of all the cancer types illustrated, there is very little variation for the comparison of *Staphylococcus* read pairs to *Propionibacterium* read pairs (Fig. 3), reinforcing that these two species are likely co-contaminants. Therefore, we conclude that *Pseudomonas* read pairs result from either separate contamination for which a source could not be identified or from a biologically relevant source like the stomach adenocarcinoma microbiome.

To determine if removal of these contaminants from our dataset would reduce bacterial diversity within each cancer type, alpha diversity was calculated before and after applying a filter to remove the contamination identified above. For filtering, all *S. epidermidis* and *Propionibacterium* read pairs were removed. Additionally, *Mycobacterium* read pairs were removed from the OV and GBM datasets and *Ralstonia* read pairs were removed from the AML dataset. After subsampling all cancer types to the minimum number of reads in the smallest cancer type, the numbers of OTUs were counted (Additional file 6: Figure S5). KIRC had the highest amount of diversity across the cancer types, while AML had the lowest amount of diversity (Additional file 6: Figure S5). It does not appear that filtering for these potential contaminants substantially altered the alpha diversity of any cancer type (Additional file 6: Figure S5).

## Discussion

### Lack of evidence supporting contamination as origin of some dominant bacterial species

Through this comprehensive evaluation of the bacterial taxa present in the Illumina paired-end reads from an early release of TCGA data, we describe the presence of bacteria associated with specific cancer types. Many researchers suggest removing reads from commonly contaminating taxa. However, this poses numerous problems, most notably that you introduce a bias. It also assumes that contamination sources are static, when they are actually fluid. For both technical and practical reasons, there is no negative control for sequencing. With this in mind, all of the data were processed and analyzed in the same manner. However, we recognize that the GBM samples can serve as a quasi-negative control since a brain tumor is unlikely to have resident bacteria due to the blood-brain barrier. The only bacterial reads found to be substantially associated with GBM samples were of *Mycobacterium* origin, which was deemed to be a sequence center-level contamination issue.

Upon conclusion of the various tests and proxies used to examine bacterial contamination of the samples, the presence of *Acinetobacter* and *Pseudomonas* spp. in AML and *Pseudomonas* spp. in STAD could not be attributed to contamination. Regardless of the measures taken to associate metadata with these bacteria, no associations between the collection centers, plates, sequencing centers, etc. were found. We also examined the sequencing projects that occurred at the British Columbia Genome Sequencing Center (BCGSC), which sequenced the AML and STAD samples. We could not find *Pseudomonas* spp. or *Acinetobacter* spp. sequencing projects ongoing at the approximate time the samples may have been sequenced, and we did not find many bacterial sequencing projects there, in general. This lends support to the *Pseudomonas*-like and *Acinetobacter*-like sequencing reads being biologically relevant. In the STAD dataset, samples were collected from two different collection centers, and while there was no correlation between the bacteria present and collection centers for most samples, a small subset of samples from Asterand had an increased abundance of *Pseudomonas*-like read pairs. As AML and STAD samples were only sequenced at BCGSC in this release of TCGA data, no further conclusions based on sequencing center could be made.

However, there are factors that complicate these analyses. Experimental methods for extracting DNA and RNA can confound these results, as these samples were collected for the primary purpose of analyzing the cancer genome and not studying bacteria. Some DNA extraction methods are preferred for breaking bacterial cell walls, meaning that samples undergoing methods that are more efficient at breaking the bacterial cell wall, such as bead beating, could have a higher bacterial yield than those extracted with other methods. Another complication of this analysis is that all of the AML samples were collected from a single center and sequenced at a single center. Therefore, there were no samples from an alternate collection center or sequencing center to use as a comparison, at least in this release of TCGA data. Additionally, the AML and STAD datasets were poly-A-selected RNA-Seq data, which will impact the bacteria identified by these analyses as discussed in the “Background” section. One might assume that this poly-A-selected RNA-Seq data is devoid of bacterial mRNA; however, we have observed in multiple projects that this is not the case. In terms of identifying cancer-related bacteria in poly-A sequencing, we only identified 142 *H. pylori* read pairs across the entire TCGA dataset (MicroView). However, we have been able to identify many more read pairs attributed to *H. pylori* in other poly-A-selected transcriptomics data generated by our group (data not shown). Therefore, in the case of TCGA data, we believe the lack of *H. pylori* read pairs was not caused by poly-A selection, but other factors (e.g., samples being collected from individuals who did not have an active *H. pylori* infection). An additional example of identifying bacteria in poly-A-selected data is a project on the fruit fly *Drosophila ananassae* and its *Wolbachia* endosymbiont (SRA Project SRP061993). A total RNA library contained 193,612 *Wolbachia* reads and 8,889,348 fruit fly reads that mapped while a poly-A-enriched RNA library contained 1923 *Wolbachia* reads and 9,318,954 fruit fly reads that mapped. This indicates that poly-A-selected RNA libraries contain bacterial reads that can be used to examine the microbiome. However, it is likely that they do not provide a complete, unbiased picture of the bacterial transcripts present in the sample.

### Pitfalls of explorations of public sequence data

The 2003 Fort Lauderdale agreement on “Sharing Data from Large-scale Biological Research Projects” and it predecessor, the 1996 Bermuda Principles, laid out foundations for the sharing of biological data, most notably genomics data. Combined with ever-evolving policies from funding agencies that support these data sharing ideals, vast amounts of data are publicly available for secondary uses. While data is frequently deposited, it is less frequently used for such secondary analyses. Our group uses such data to identify previously undetected interdomain lateral gene transfers [30, 37], while other notable examples of secondary data use involve studies on identifying biomarkers for preeclampsia [38], cell surface targets of medulloblastoma [39], and factors associated with organ transplant rejection [40, 41]. When possible, it is best to merge multiple datasets and find characteristics in common across those datasets, lending support to those conclusions. Here, we use such common results to identify common contaminants in some datasets, like staphylococci and propionibacteria, which are frequently found across all datasets and in a similar relative proportion to one another.

While such cross dataset analysis is possible when numerous datasets can be aggregated, it is not always possible. In such cases where a limited number of datasets of a given type are available, it can be informative to evaluate potential batch effects or other correlations that can be linked via the metadata. In the case of our analysis, this revealed a correlation between sequencing center and *M. tuberculosis* complex in the OV and GBM samples and a correlation between plates and *Ralstonia* spp. in AML samples.

It is also important to recognize the limitations of such secondary data analyses, especially when data is used to address an alternate question. For example, much of the TCGA data is RNA-Seq or whole exome data collected following selective capture of certain nucleic acids such that bacterial profiles examined may be incomplete. A recent study showed that microbial communities in the saliva were not biased in exome sequence data compared to whole genome sequence data [42]. However, it is not clear how widespread this observation may be, and while some bacterial associations can be identified, others may be lost due to the methods used. In the release of the TCGA that we used, there were no samples that had whole genome sequencing (WGS), whole exome sequencing (WXS), and RNA-Seq data. One GBM sample had DNA and RNA extracted, and both sequence types possessed bacterial read pairs. The RNA data had 208 bacterial read pairs, primarily comprised of *Enterobacteriaceae*. The DNA sample contained 1421 bacterial read pairs with one third of the read pairs contributed by *P. acnes.* Both of these bacteria we attribute as contamination, and otherwise, the bacterial taxa identified were very similar. This is not surprising given that GBM samples are not expected to have bacterial sequences present, as noted above. We also compared one STAD RNA-Seq sample in this dataset with WGS and WXS data from the same sample from a subsequent TCGA data release. While there were a few minor differences between WGS and WXS, the bacterial taxa identified were very similar between the two sequencing types (Additional file 7: Figure S6). There were almost equal proportions of *Firmicutes*, *Fusobacteria*, and *Bacteroidetes* when compared between the WGS and WXS results. The specificity of the taxonomic assignments was also very similar between WGS and WXS. However, there were large differences in bacterial diversity when comparing RNA-Seq data to WGS or WXS, with RNA-Seq data having an enrichment of *Proteobacteria* and lacking a significant number of read pairs from other phyla (Additional file 7: Figure S6). While the WGS and WXS data also contained bacterial read pairs from *Proteobacteria*, these read pairs were in the minority (Additional file 7: Figure S6).

There may also be a difference in the types of genes found from each bacterial taxa (Additional file 8: Table S1). GBM and OV were the only cancer types investigated where DNA sequencing was completed, which is reflected by the increased percentage of GBM read pairs that aligned to protein coding regions (CDSs) compared to the other cancer types. The other eight cancer types, which were RNA-Seq datasets, had 68–90% of the bacterial read pairs aligning to rRNA, with only 3–31% of the reads aligning to coding sequences (Additional file 8: Table S1). This likely reflects the normal composition of these nucleic acids, with total RNA dominated by rRNA while DNA has a composition reflecting the entire genome.

In the cases of the *Acinetobacter* association with AML and the *Pseudomonas* association with STAD, we cannot distinguish between contamination and a biologically relevant association. Since samples are not available for follow-up studies, prospective samples will need to be collected to test these hypotheses in the future. Despite the need for further experimentation, analyses of large public data repositories such as this one, provide an important and cost-effective opportunity to develop numerous new hypotheses that have the potential to challenge dogma.

## Conclusions

A more thorough evaluation of publicly available sequence data with a microbiome-focused analysis may be fruitful. However, methods to examine contamination and batch effects should be used. In the case of public sequencing data, it can sometimes be very difficult to determine experimental protocol and determine what bacterial taxa in the samples may be due to batch effects from experimental protocols and what bacteria are actually in the samples. This suggests that more metadata, as well as better structured metadata, may be necessary to ensure these datasets can be used more successfully in secondary analyses. Based on our analysis, we suggest that nucleic acid extraction method, collection site, sequencing site, tissue type, sequence type, library type, library methods, and antibiotic treatment must be included in a manner that is easy to retrieve. In addition, researchers acutely need methods to track other nucleic-acid-based projects simultaneously occurring in the source sites, including the tissue source site, the sequencing center, and any other collection sites.

## Methods

### Pipeline for identifying bacterial reads and taxonomic assignments

The pipeline for executing alignments with BWA version 0.5.9-r16 and discarding low complexity and duplicate reads with PRINSEQ version 0.20.3 [43] has previously been described [30]. Briefly, alignments were constructed to all complete bacterial genomes in RefSeq as well as the human reference genome, hg19, using BWA ALN version 0.5.9-r16 [44]. Other alignment algorithms including BWA MEM and MOSAIK were tested on other datasets. BWA MEM is more sensitive, but less specific, unless the database includes the host and all microbes, which was not possible here.

After aligning the read pairs to all of the references, pairs of reads where neither read matched hg19, but both matched bacteria were selected for further analysis. OTUs were calculated for each read pair using the BWA results [30, 45]. Each individual read in the read pair was given the taxonomic assignment of its best match, or the aggregate common taxonomy of all of its best matches. The read pair was then assigned an OTU by comparing the two taxonomic assignments of the reads in the pair and using the common taxonomy (Additional file 1: Figure S1). Reads were attributed to the bacterial portion of the microbiome when both reads had an OTU suggesting “bacteria.”

### Construction of MicroView interactive data browser

Taxonomic assignments and metadata for approximately 36 million de-identified reads were loaded into a PostgreSQL relational database. Materialized views were then created to summarize read counts by every observed combination of metadata field values and taxonomic assignment. Components of the LGTView user interface [30] were combined with a new set of Python CGI scripts to create MicroView, an interactive interface that allows a user to drill down into the 36 million reads and 9 cancer types using any combination of the available metadata fields: cancer type, plate, sequencing center, tissue source site, SRA run ID, sample/vial, analyte, taxonomic assignment, and anonymized sample id.

### Heat map preparation

The 36 million putative microbiome reads were parsed into their most specific taxonomic category to the family level with the exception of reads classified as arising from *Ralstonia*, *Mycobacterium*, *Propionibacterium*, *Staphylococcus*, *Acinetobacter*, or *Pseudomonas*, where a genus level assignment was used. The log_10_-transformed read counts per sample per taxonomic category were normalized to the number of total reads per sample, multiplied by 100,000 and plotted in a heat map (Fig. 2). A sample was defined as a specific nucleic acid sequenced by a specific center. Therefore, DNA and RNA would be considered different samples as would the same DNA that was sequenced by two different sequencing centers. Only taxa with >20 reads per one million total reads are visualized. Samples were clustered using agglomerative hierarchical clustering in the heatmap.2 function of GPLOTS version 3.0.1 running in R version 3.3.1. Each sample was color-coded by the associated metadata, which is organized in the key by the numeric code assigned by the TCGA.

### BLASTN searches for *Mycobacterium*

Searches to confirm *M. tuberculosis* complex read pairs were completed using the NCBI tool BLASTN [46] with the default settings. All of the OV and GBM read pairs with matches identified by BWA to *M. tuberculosis complex* were searched against the NT database. Figures representing the bacterial proportions in cancer and normal samples were created using Krona [35].

### Log ratio calculations comparing *Mycobacterium* read pairs to total read pairs

The log_10_ ratio comparing the number of *Mycobacterium* spp. read pairs per run to the total number of reads per run was calculated as the log_10_ of the number of *Mycobacterium* spp. read pairs in each run divided by the total number of read pairs in each run. The ratio comparing the number of *Mycobacterium* spp. read pairs per sample to the total number of read pairs per sample was calculated as the log_10_ of the number of *Mycobacterium* spp. read pairs in each sample divided by the total number of read pairs in each sample.

### Log ratio calculations comparing select bacterial read pairs

*Mycobacterium* (M), *Acinetobacter* (A), and *Pseudomonas* (Ps) read pairs were defined as microbiome-associated bacteria. ﻿*S*. *epidermidis* (S), *Ralstonia* (R), and *Propionibacterium* (P) read pairs were defined as potential contaminant bacteria. The log_10_ ratios comparing the microbiome-associated bacteria to the potential contaminant bacteria were calculated by taking the log_10_ (number of microbiome-associated bacteria read pairs/number of contaminant bacteria read pairs). Subsequently, *S. epidermidis* and *Propionibacterium* (S + P) were grouped together after an initial comparison found them to frequently have the same relative proportions. They are both commonly found on the human skin and may arrive in the samples in the same manner. The average of each log_10_-transformed ratio across all the cancer types or across all of the samples was calculated and illustrated by a black horizontal line. As not all of the samples had read pairs with a particular bacterial OTU, some of the comparisons would not be calculated. Ratios where the numerator was 0 were assigned an arbitrary value of −7 for that ratio, ratios where both the numerator and denominator were 0 were assigned an arbitrary value of 0, and ratios where the denominator was 0 were assigned an arbitrary value of 7.

### Alpha diversity calculations

Alpha diversity was calculated by counting the total number of OTUs present from each cancer type before and after filtering for potential contaminants. Due to the large discrepancies in the number of bacterial read pairs across all of the cancer types, subsampling was necessary. Therefore, all cancer types were subsampled to the number of bacterial read pairs from GBM, which had the fewest bacterial read pairs. Subsampling was done with Mothur [47] version 1.36.1, using the sub.sample function with default settings. From this, the total number of OTUs present within each cancer type were counted and plotted. In the filtered dataset, all *S. epidermidis* and *Propionibacterium* read pairs were removed. In addition, all *Ralstonia* read pairs were removed from the AML dataset and all *Mycobacterium* read pairs were removed from the OV and GBM datasets.

### Gene feature calculations

The mappings of each bacterial read pair to all of the complete bacterial genomes in RefSeq were combined with genome annotations to determine the gene features for each bacterial read pair alignment.

## Additional files

Additional file 1: Figure S1. This schematic illustrates how taxonomy was assigned to the bacterial read pairs. Reads not aligning to the human genome reference were aligned to all complete bacterial genomes in RefSeq using BWA. After the alignment is complete, our pipeline determines which reads have aligned to each of the bacterial reference genomes. An approach is used to identify a taxonomic assignment that encompasses all of the operational taxonomic units of all of the matches for a read. Then, the assignments for read pairs are combined such that the most specific taxonomic assignment is used as the bacterial OTU as illustrated using this previously described scheme [30]. (PDF 521 kb)
Additional file 2: This file is a tab-delimited text file of all TCGA read pairs and their corresponding metadata and bacterial taxonomic assignments. Comments are denoted by a #. Abbreviations for sequencing center and tissue source site are as follows: BCGSC—British Columbia Genome Sequencing Center; Broad—The Broad Institute of MIT and Harvard; Fox Chase—Fox Chase Cancer Center; MSKCC—Memorial Sloan Kettering Cancer Center; UCSF—University of California San Francisco; UNC—University of North Carolina; Wash Univ St. Louis—Washington University in St. Louis. This file is available at Dryad with the following link: http://dx.doi.org/10.5061/dryad.96584. (GZ 259 kb)
Additional file 3: Figure S2. These histograms illustrate the variation in counts of bacterial taxa per sequencing run across cancer types. The number of bacterial read pairs per run is shown for tumor and normal samples for all cancer types investigated. (PDF 919 kb)
Additional file 4: Figure S3. The proportions of bacterial read pairs for both tumor (panel A) and normal-matched tissue (panel B) for glioblastoma multiforme (GBM) are illustrated. The GBM dataset had about the same number of bacterial read pairs per sample for both the 78 normal and the 78 tumor samples. Both sample types had about a third of the read pairs from *Ralstonia picketti* with low levels of read pairs from *Enterobacteriaceae*, *Pseudomonas aeruginosa*, and *Mycobacterium tuberculosis* complex. (PDF 841 kb)
Additional file 5: Figure S4. The log_10_-transformed ratios of bacterial counts by patient were calculated for the breast cancer data as described in Fig. 3. Samples were color-coded by sample type in panel A and by plate in panel B. All of the samples were sequenced at the same center. In panel B, the samples segregate by plate for ratios containing *Ralstonia* spp., suggesting that these plates may have been contaminated. (PDF 329 kb)
Additional file 6: Figure S5. Alpha diversity for all cancer types was compared pre- and post-filtering. The total number of OTUs before and after filtering for potential contaminant bacterial read pairs is plotted for each cancer type. Subsampling was done to accurately compare the large datasets, like AML and STAD to the smallest dataset, GBM. No major differences in alpha diversity were observed after filtering. (PDF 118 kb)
Additional file 7: Figure S6. The proportion of bacterial read pairs for RNA-Seq (panel A), whole genome sequencing (WGS, panel B), and whole exome sequencing (WXS, panel C) for an individual stomach adenocarcinoma (STAD) sample are illustrated. The RNA-Seq data shows an enrichment of *Proteobacteria* that have low proportions in the WGS and WXS data. WGS and WXS sequencing methods resulted in a more diverse collection of bacterial read pairs with only minor differences between the two methods. (PDF 445 kb)
Additional file 8: Table S1. Types of gene features represented by the bacterial read pairs of each cancer type. (DOC 54 kb)

## Abbreviations

AML: Acute myeloid leukemia
BCGSC: British Columbia Genome Sequencing Center
BRCA: Breast cancer
Broad: The Broad Institute of MIT and Harvard
BWA: Burrows-Wheeler aligner
EBV: Epstein-Barr virus
Fox Chase: Fox Chase Cancer Center
GBM: Glioblastoma multiforme
HBV: Hepatitis B virus
HIV: Human immunodeficiency virus
HPV: Human papillomavirus
KIRC: Kidney clear cell carcinoma
KIRP: Kidney papillary carcinoma
LGT: Lateral gene transfer
LUAD: Lung adenocarcinoma
LUSC: Lung squamous cell carcinoma
MALT: Mucosa-associated lymphoid tissue
MSKCC: Memorial Sloan Kettering Cancer Center
OTU: Operational taxonomic unit
OV: Ovarian serous cystadenocarcinoma
RNA-Seq: RNA sequencing
SRA: Sequence Read Archive
STAD: Stomach adenocarcinoma
TCGA: The Cancer Genome Atlas
UCSF: University of California San Francisco
UNC: University of North Carolina
Wash Univ St. Louis: Washington University in St. Louis
WGA: Whole genome amplified
WGS: Whole genome sequencing
WXS: Whole exome sequencing
