# Supplementary figures and images for: Distinguishing potential bacteria-tumor associations from contamination in a secondary data analysis of public cancer genome sequence data
Source: Microbiome. 2017 Jan 25;5:9. doi: 10.1186/s40168-016-0224-8 (PMC5264480; doi:10.1186/s40168-016-0224-8)

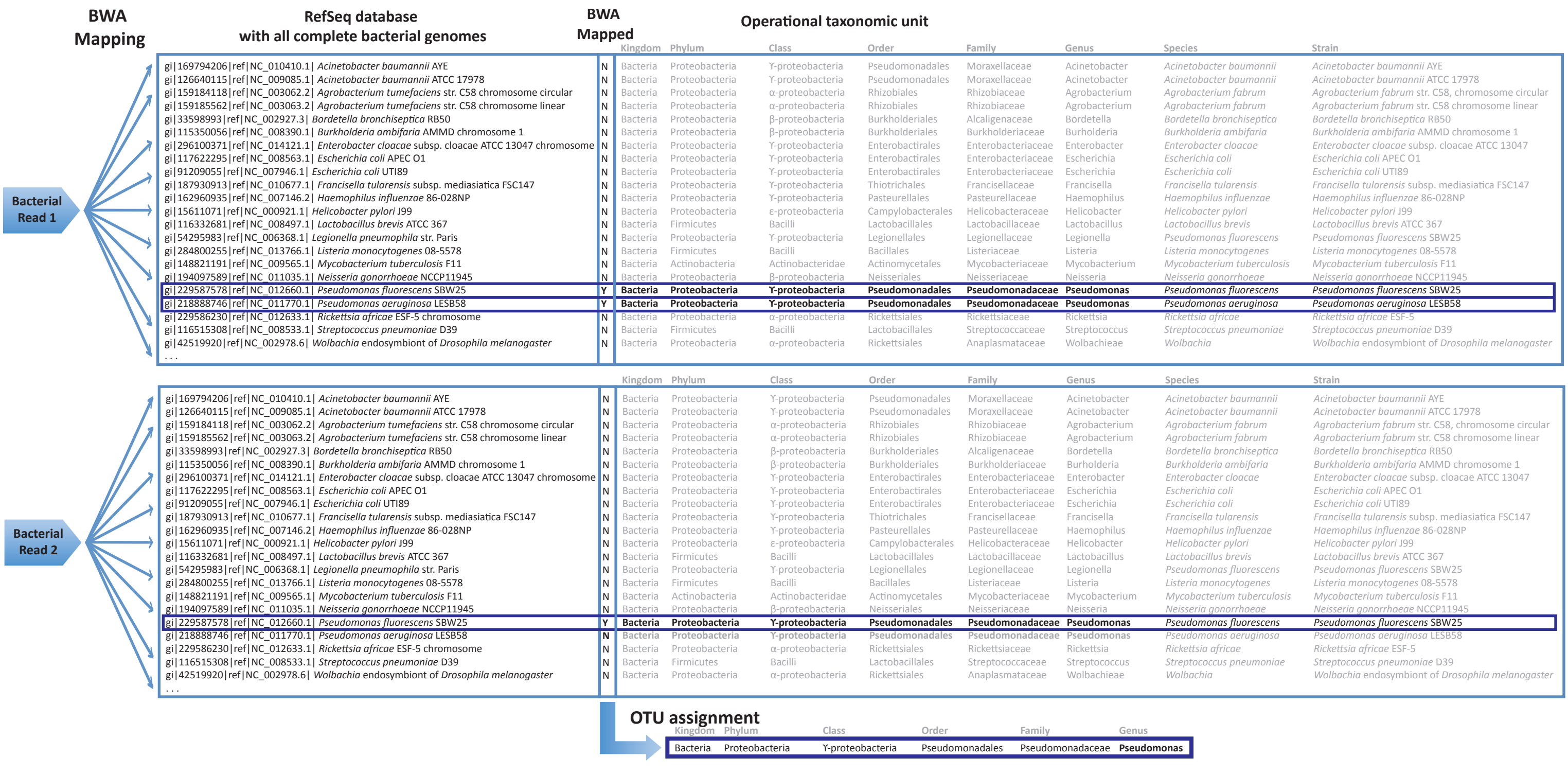

Supplement: Additional file 1: Figure S1. — This schematic illustrates how taxonomy was assigned to the bacterial read pairs. Reads not aligning to the human genome reference were aligned to all complete bacterial genomes in RefSeq using BWA. After the alignment is complete, our pipeline determines which reads have aligned to each of the bacterial reference genomes. An approach is used to identify a taxonomic assignment that encompasses all of the operational taxonomic units of all of the matches for a read. Then, the assignments for read pairs are combined such that the most specific taxonomic assignment is used as the bacterial OTU as illustrated using this previously described scheme [30]. (PDF 521 kb) [file 40168_2016_224_MOESM1_ESM.pdf]

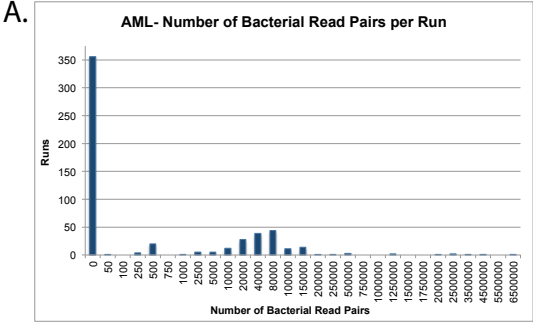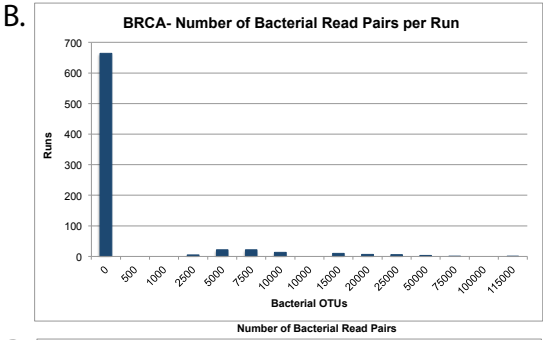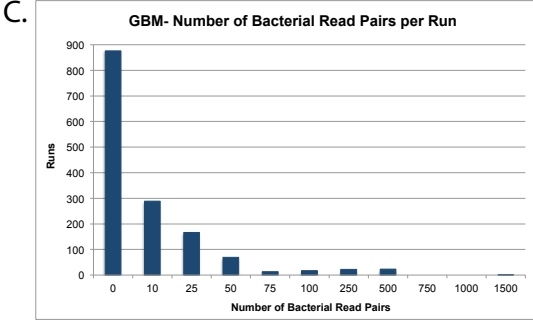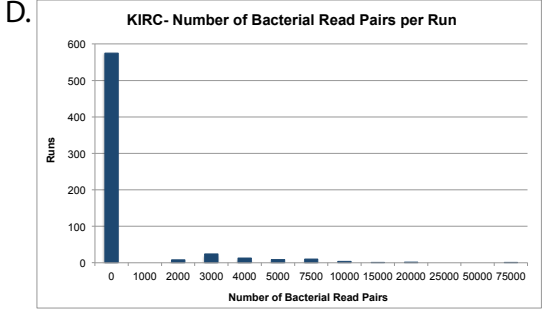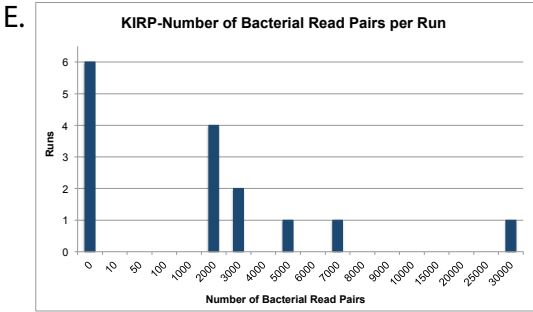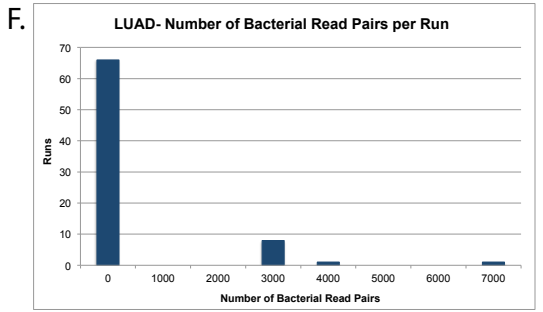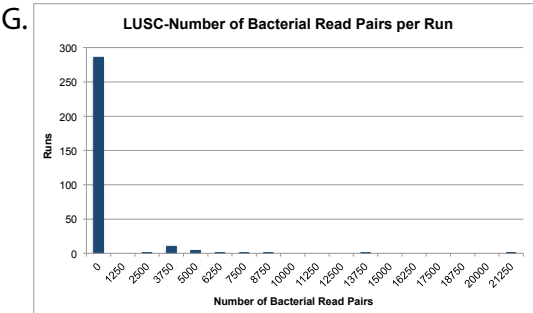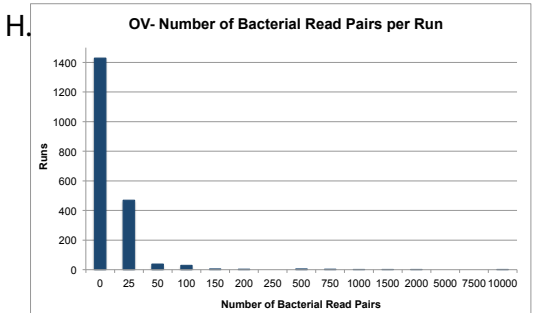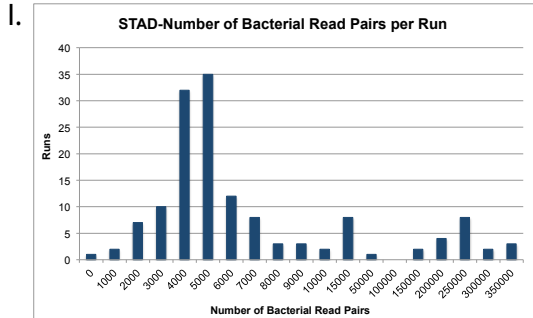

Supplement: Additional file 3: Figure S2. — These histograms illustrate the variation in counts of bacterial taxa per sequencing run across cancer types. The number of bacterial read pairs per run is shown for tumor and normal samples for all cancer types investigated. (PDF 919 kb) [file 40168_2016_224_MOESM3_ESM.pdf]

A.

## GBM Normal

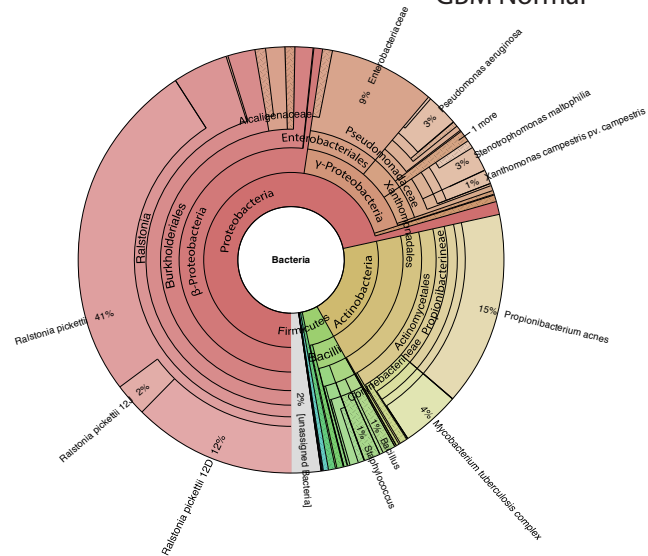

B.

## GBM Tumor

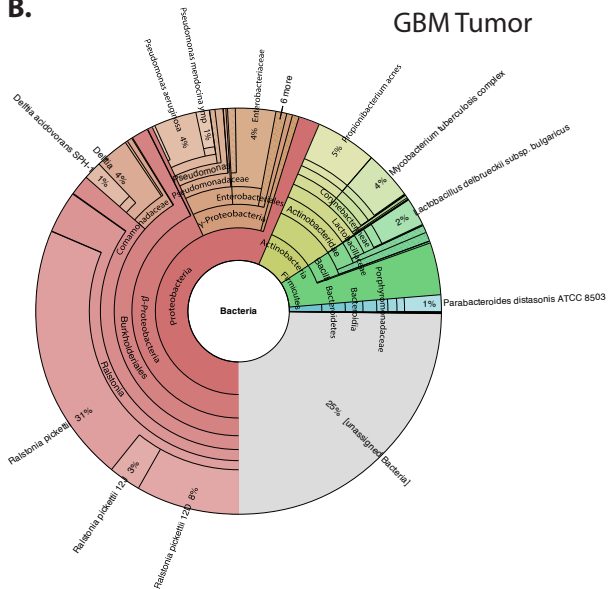

Supplement: Additional file 4: Figure S3. — The proportions of bacterial read pairs for both tumor (panel A) and normal-matched tissue (panel B) for glioblastoma multiforme (GBM) are illustrated. The GBM dataset had about the same number of bacterial read pairs per sample for both the 78 normal and the 78 tumor samples. Both sample types had about a third of the read pairs from Ralstonia picketti with low levels of read pairs from Enterobacteriaceae, Pseudomonas aeruginosa, and Mycobacterium tuberculosis complex. (PDF 841 kb) [file 40168_2016_224_MOESM4_ESM.pdf]

A

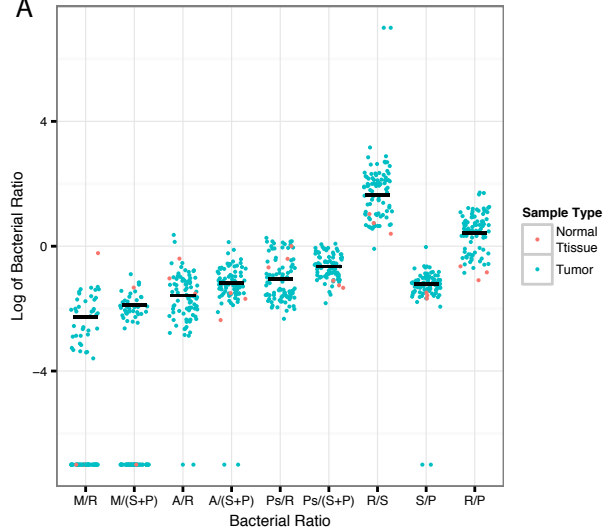

|               |    |    |   |   |   |   |   |   |   |
|---------------|----|----|---|---|---|---|---|---|---|
| Value of +7 = | 0  | 0  | 0 | 0 | 0 | 0 | 2 | 0 | 0 |
| Value of 0 =  | 0  | 0  | 0 | 0 | 0 | 0 | 0 | 0 | 0 |
| Value of -7 = | 50 | 50 | 2 | 2 | 0 | 0 | 0 | 2 | 0 |

B

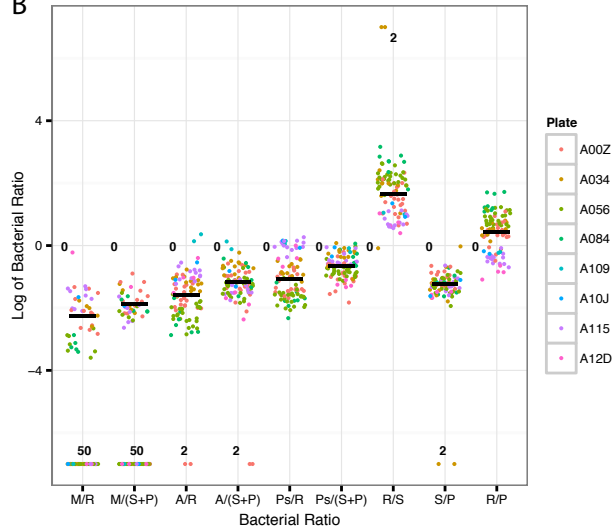

|               |    |    |   |   |   |   |   |   |   |
|---------------|----|----|---|---|---|---|---|---|---|
| Value of +7 = | 0  | 0  | 0 | 0 | 0 | 0 | 2 | 0 | 0 |
| Value of 0 =  | 0  | 0  | 0 | 0 | 0 | 0 | 0 | 0 | 0 |
| Value of -7 = | 50 | 50 | 2 | 2 | 0 | 0 | 0 | 2 | 0 |

Supplement: Additional file 5: Figure S4. — The log10-transformed ratios of bacterial counts by patient were calculated for the breast cancer data as described in Fig. 3. Samples were color-coded by sample type in panel A and by plate in panel B. All of the samples were sequenced at the same center. In panel B, the samples segregate by plate for ratios containing Ralstonia spp., suggesting that these plates may have been contaminated. (PDF 329 kb) [file 40168_2016_224_MOESM5_ESM.pdf]

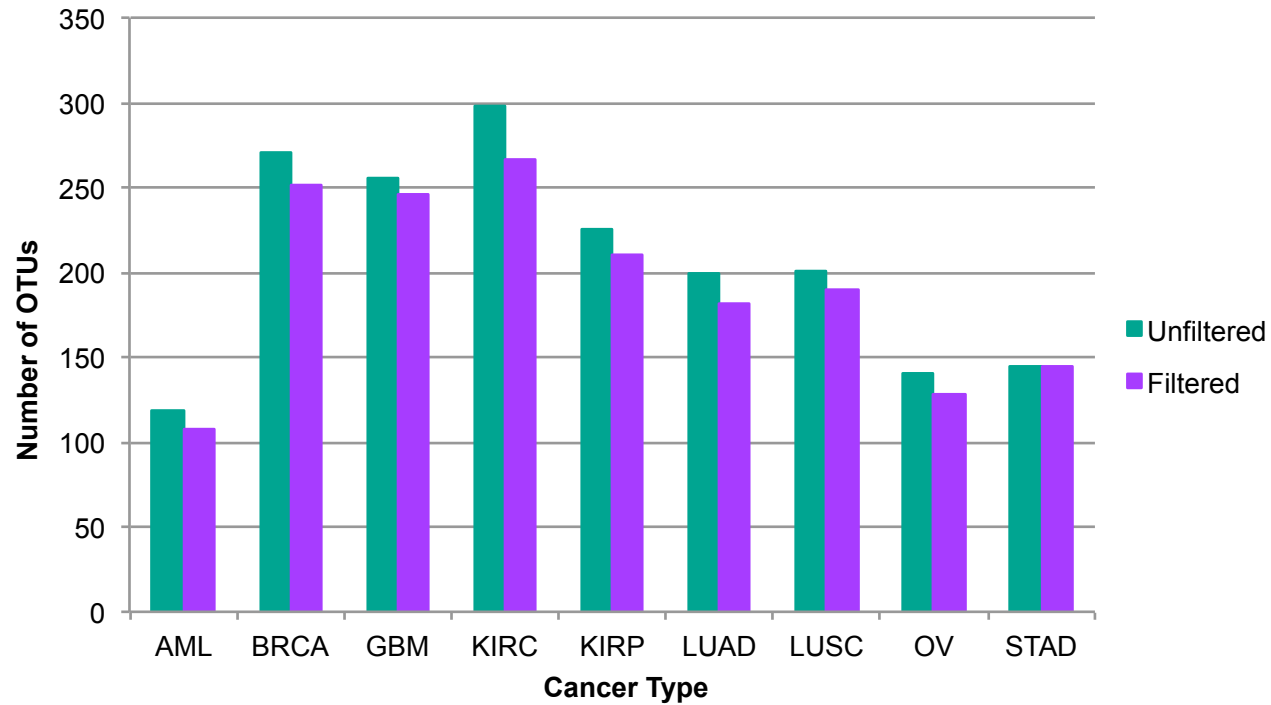

Supplement: Additional file 6: Figure S5. — Alpha diversity for all cancer types was compared pre- and post-filtering. The total number of OTUs before and after filtering for potential contaminant bacterial read pairs is plotted for each cancer type. Subsampling was done to accurately compare the large datasets, like AML and STAD to the smallest dataset, GBM. No major differences in alpha diversity were observed after filtering. (PDF 118 kb) [file 40168_2016_224_MOESM6_ESM.pdf]

A. RNA-Seq

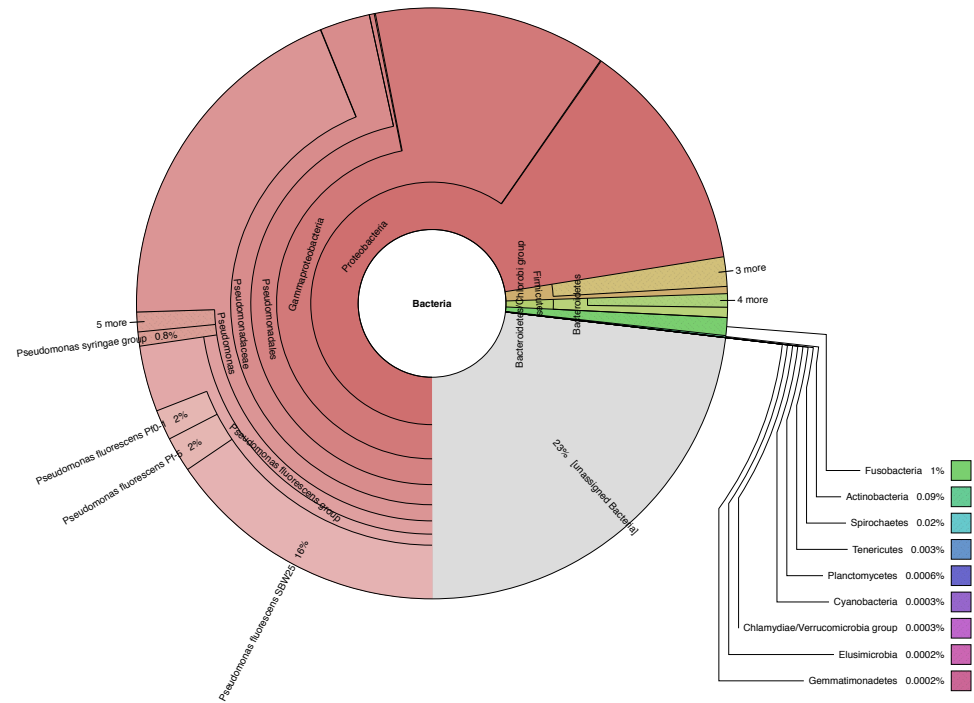

B. WGS

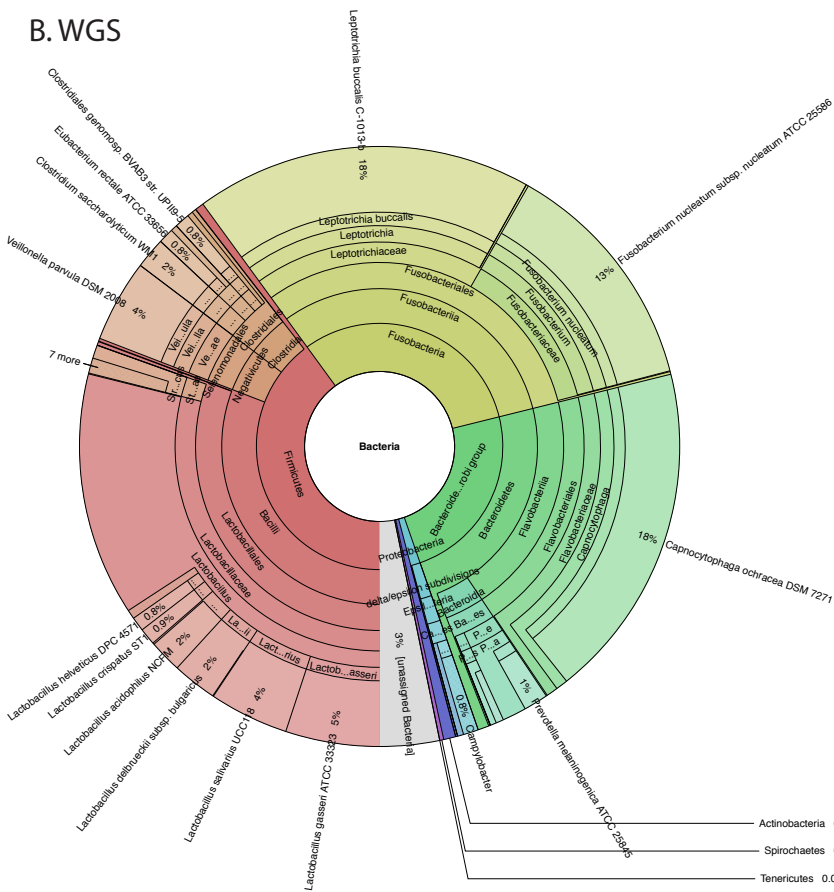

C. WXS

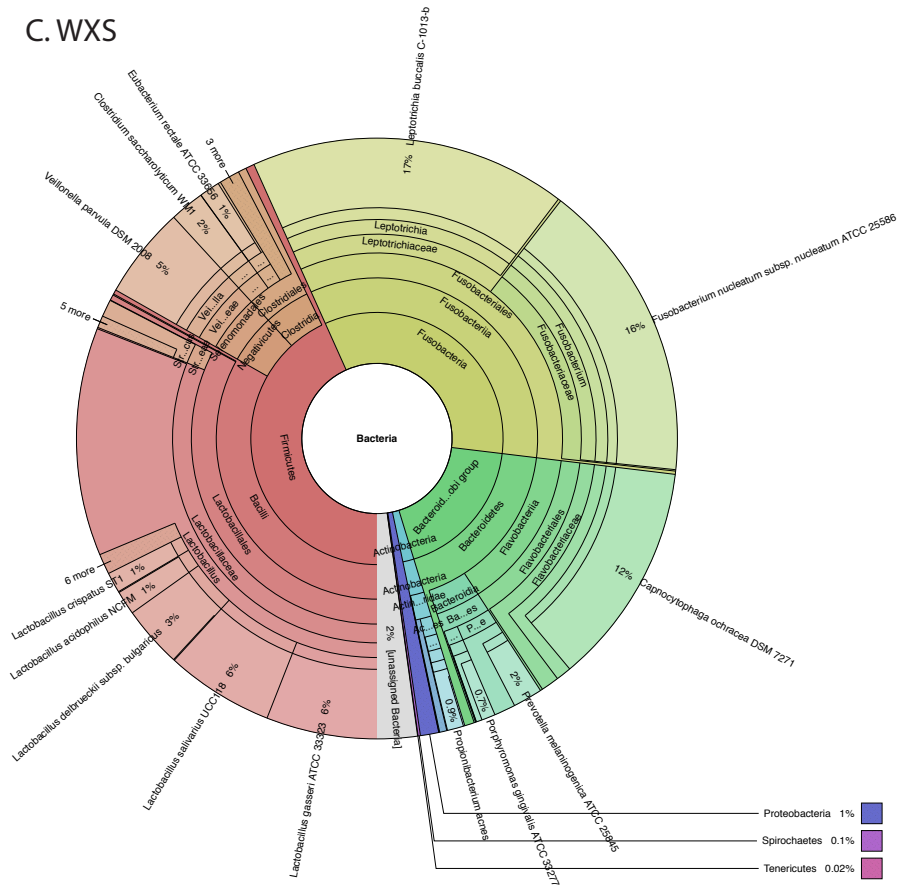

Supplement: Additional file 7: Figure S6. — The proportion of bacterial read pairs for RNA-Seq (panel A), whole genome sequencing (WGS, panel B), and whole exome sequencing (WXS, panel C) for an individual stomach adenocarcinoma (STAD) sample are illustrated. The RNA-Seq data shows an enrichment of Proteobacteria that have low proportions in the WGS and WXS data. WGS and WXS sequencing methods resulted in a more diverse collection of bacterial read pairs with only minor differences between the two methods. (PDF 445 kb) [file 40168_2016_224_MOESM7_ESM.pdf]
