# Supplementary material for: Distinguishing potential bacteria-tumor associations from contamination in a secondary data analysis of public cancer genome sequence data
Source: Microbiome. 2017 Jan 25;5:9. doi: 10.1186/s40168-016-0224-8 (PMC5264480; doi:10.1186/s40168-016-0224-8)
Supplement: Additional file 8: Table S1. — Types of gene features represented by the bacterial read pairs of each cancer type. (DOC 54 kb) [file 40168_2016_224_MOESM8_ESM.doc]

| **Feature** | **AML** | | **BRCA** | | **GBM** | | **KIRC** | | **KIRP** | | **LUAD** | | **LUSC** | | **OV** | | **STAD** | |
| --- | --- | --- | --- | --- | --- | --- | --- | --- | --- | --- | --- | --- | --- | --- | --- | --- | --- | --- |
|  | **Read Pairs** | **%** | **Read Pairs** | **%** | **Read Pairs** | **%** | **Read Pairs** | **%** | **Read Pairs** | **%** | **Read Pairs** | **%** | **Read Pairs** | **%** | **Read Pairs** | **%** | **Read Pairs** | **%** |
| **CDS** | 2.09x10^8^ | 3.29 | 2.03x10^7^ | 21.48 | 210,687 | 73.49 | 7.47x10^6^ | 13.92 | 947,437 | 9.65 | 1.14x10^6^ | 31.17 | 2.72x10^6^ | 21.71 | 599,995 | 26.54 | 5.06x10^7^ | 12.16 |
| **Gene** | 3,687 | 0.00 | 1,654 | 0.00 | 14 | 0.00 | 317 | 0.00 | 24 | 0.00 | 49 | 0.00 | 152 | 0.00 | 1 | 0.00 | 1,211 | 0.00 |
| **Mature peptide** | 19,601 | 0.00 | 43,857 | 0.05 | 2,397 | 0.84 | 23,033 | 0.04 | 3,058 | 0.03 | 4,169 | 0.11 | 8,363 | 0.07 | 5,342 | 0.24 | 101,516 | 0.02 |
| **Misc. RNA** | 124,229 | 0.00 | 15,318 | 0.02 | 1,068 | 0.37 | 5,806 | 0.01 | 750 | 0.01 | 940 | 0.03 | 2,212 | 0.02 | 414 | 0.02 | 154,427 | 0.04 |
| **Noncoding RNA** | 85,558 | 0.00 | 7,161 | 0.01 | 234 | 0.08 | 2,653 | 0.00 | 402 | 0.00 | 286 | 0.01 | 1,027 | 0.01 | 153 | 0.01 | 157,711 | 0.04 |
| **Miscellaneous** | 312 | 0.00 | 30 | 0.00 | 0 | 0.00 | 24 | 0.00 | 12 | 0.00 | 1 | 0.00 | 4 | 0.00 | 0 | 0.00 | 3,102 | 0.00 |
| **rRNA** | 6.16x10^9^ | 96.68 | 7.39x10^7^ | 78.18 | 71,042 | 24.78 | 4.61x10^7^ | 85.94 | 8.86x10^6^ | 90.27 | 2.52x10^6^ | 68.53 | 9.78x10^6^ | 78.00 | 1.65x10^6^ | 73.12 | 3.64x10^8^ | 87.56 |
| **Signal peptide** | 34,262 | 0.00 | 2,051 | 0.00 | 244 | 0.09 | 932 | 0.00 | 22 | 0.00 | 36 | 0.00 | 189 | 0.00 | 84 | 0.00 | 27,243 | 0.01 |
| **tmRNA** | 872 | 0.00 | 883 | 0.00 | 2 | 0.00 | 294 | 0.00 | 30 | 0.00 | 22 | 0.00 | 63 | 0.00 | 0 | 0.00 | 681 | 0.00 |
| **tRNA** | 1.28x10^6^ | 0.00 | 248,140 | 0.26 | 993 | 0.35 | 42,038 | 0.08 | 3,736 | 0.04 | 5,569 | 0.15 | 24,320 | 0.19 | 1,650 | 0.07 | 709,486 | 0.17 |
| **Sum** | 6.37x10^9^ |  | 9.46x10^7^ |  | 286,681 |  | 5.36x10^7^ |  | 9.82x10^6^ |  | 3.68x10^6^ |  | 1.25x10^7^ |  | 2.26x10^6^ |  | 4.16x10^8^ |  |
